# Synovial bone sialoprotein indicates aseptic failure in total joint arthroplasty

**DOI:** 10.1186/s13018-020-01718-2

**Published:** 2020-05-27

**Authors:** André Busch, Marcus Jäger, Florian Dittrich, Alexander Wegner, Stefan Landgraeber, Marcel Haversath

**Affiliations:** 1 Department of Orthopaedics, Trauma and Reconstructive Surgery, St. Marien-Hospital Mülheim a.d. Ruhr, 45468 Mülheim an der Ruhr, Germany; 2 Orthopaedics and Trauma Surgery, University of Duisburg – Essen, 47057 Duisburg, Germany; 3 Department of Orthopaedics, University of Saarland, 66123 Saarbrücken, Germany; 4 Department of Orthopaedics, St. Vinzenz-Krankenhaus, Schloßstraße 85, 40477 Düsseldorf, Germany

## Abstract

**Background:**

Until today, a reliable diagnostic discrimination between periprosthetic joint infections (PJI) and aseptic failure (AF) after total joint arthroplasty (TJA) remains challenging. Nearly all recent research focused on synovial markers to be elevated in PJI rather than in AF patients. In this study, synovial bone sialoprotein (sBSP) was investigated in PJI and AF arthroplasty patients before revision surgery.

**Methods:**

sBSP and C-reactive protein (CRP) were determined in synovial fluid samples of PJI (*n* = 13) patients fulfilling the MSIS criteria and AF (*n* = 25) patients. Beside descriptive analysis and comparison, computed statistics determined the area under the receiver operating characteristics curve (AUC) to evaluate the discrimination ability of the tested synovial markers.

**Results:**

In patients with PJI according to the MSIS criteria, mean sBSP was significantly lower: 14.8 ng/ml (95% CI 5.5-24.1) vs. 38.2 ng/ml in the AF group (95% CI 31.1-45.3), *p* ≤ 0.001. Conversely, mean sCRP was significantly higher in PJI patients: 8.4 μg/ml (95% CI 0-17.2) vs. 1.8 μg/ml in the AF group (95% CI 0.9-2.8), *p* = 0.032. The AUC of sCRP in PJI patients was 0.71. The AUC of sBSP in AF revision arthroplasty patients was 0.83. The detection of osteolyses was not associated with higher sBSP concentrations.

**Conclusions:**

Considering the MSIS criteria, significantly higher sBSP concentrations were found in synovial fluid samples of AF compared to PJI patients. sCRP showed only fair, sBSP good discrimination potential. If it is not clear whether PJI is present or not, sBSP may be considered as an add-on synovial marker.

## Introduction

Periprosthetic joint infection (PJI) is a severe complication after total joint arthroplasty. It is the third leading cause for revision surgery in failure hip arthroplasty [1]. The 5-year incidence rate exceeds one percent following the primary procedure. Not only in the USA but worldwide, revision arthroplasty is predicted to grow considerably in the next decades. Among others, notable risk factors for the development of periprosthetic joint infections are internal comorbidities, male gender, overweight, and prolonged surgery time. The differentiation between aseptic and septic failure is crucial for surgical planning. According to the International Consensus Group, a minimum of two positive cultures of periprosthetic tissue or the presence of a sinus tract with evidence of communication to the joint or visualization of the prothesis are major criteria in the diagnosis of PJI [2]. A major problem remains that microbiological cultures still produce false negative or positive results. Besides white cell count and C-reactive protein (CRP), other, more sensitive and specific serum or synovial biomarkers are in focus of current research [3–5]. For instance, Procalcitonin and Interleukin-6, which are commonly used to evaluate inflammation processes, were investigated toward their reasonable determination in PJI diagnostics. However, they also reveal deficits in sensitivity and specificity [3]. Alpha defensin is another synovial marker that has found its way to the market with a quantitative laboratory ELISA and a qualitative quick test that is designated as an aid in the intraoperative diagnosis of PJI (Synovasure® alpha-defensin test, Zimmer Biomet). The latest meta-analysis revealed a promising diagnostic sensitivity and specificity of alpha defensin in PJI diagnostics [6]. Conversely, other authors attested a poor test sensitivity independent of the test method, quantitative or qualitative [7, 8]. Yet, there is no “gold standard” in serum or synovial fluid biomarkers for reliable diagnosis of PJI [9]. On the other side, there are no reliable synovial markers that indicate aseptic TJA failure.

Bone sialoprotein (BSP) is a glycoprotein that is only found in the extracellular matrix of bone and dentine [10]. High concentrations of BSP are located in the osteoid, the newly formed bone tissue of growing bone, which is the most common site for osteomyelitis [11]. It has been shown that BSP selectively binds to staphylococci isolated from patients suffering from osteomyelitis and septic arthritis [12]. The bacterial cell wall glycoprotein BSP-binding protein (Bbp) induces an immune response and elevated serum IgG-antibodies to Bbp were found to be related to *S. aureus* osteomyelitis of the diabetic foot [13]. Due to the bacterial binding of BSP, we speculated to detect lower synovial levels in PJI patients.

The purpose of this study was to investigate the diagnostic and prognostic value of BSP in synovial fluid for the diagnosis of aseptic failure TJA. Furthermore, synovial BSP (sBSP) was compared to the already evaluated synovial C-reactive protein (sCRP) and other than sBSP known to be enhanced in PJI patients.

## Material and methods

### Study design

This research has been approved by the IRB of the authors’ affiliated institutions.

Preoperatively, the medical history was recorded and clinical examination, laboratory values including serum CRP and joint aspiration fluid were investigated as routine diagnostic procedures in revision arthroplasty of the hip, knee, and shoulder. Furthermore, preoperative X-rays were analyzed toward manifest osteolyses by three independent observers (all orthopedic surgeons). Inclusion criteria were an adequate synovial fluid volume for laboratory marker measurements as well as full clinical and laboratory data to allow the diagnosis of PJI. Patients suffering from systemic inflammatory diseases (SID) were also included. Patients receiving antibiotics before joint aspiration and cases of early postoperative PJI (8 weeks) were excluded because of the lack of reliability in the determination of synovial and serologic markers shortly after surgery [14, 15].

The differential diagnosis between aseptic and septic was made according to the musculoskeletal infection society (MSIS) excluding the sedimentation rate as a minor criterion [16, 17].

### Patients

From July 2018 to March 2019, 38 patients received revision arthroplasty and were included in the study. Written informed consent to participate in the study was obtained prior to intervention. Tables 1 and 2 summarize the main patient characteristics.

**Table 1:** Main patient characteristics

	PJI patients (according to MSIS)			Aseptic failure of arthroplasty		
Number (*n*)	13			25		
Gender ratio (m/f)	5/8			6/19		
MSIS major criteria	12/13			-		
Affected joint						
Hip/knee/shoulder	6/6/1			14/9/2		
Radiological evidence of prosthetic loosening						
- Cup (hip)	1			4		
- Stem (hip)	4			3		
- Femoral component (knee)	3			1		
- Tibial component (knee)	4			0		
- Glenoid/glenosphere (shoulder)	1			0		
- Humeral component (shoulder)	1			3		
Osteolyses	Mild	Moderate	Severe	Mild	Moderate	Severe
	4	3	3	8	7	2
Bacteria detection in synovial fluid						
- *S. aureus*	2			-		
- *S. epidemidis*	2					
- *S. warneri*	1					
- *Klebsiella pneumoniae*	3					
- *Pseudomonas aeruginosa*	1					
- *Cutibacterium acnes*	1					
- *E. coli*	1					
- None	2					
Evidence of systemic inflammatory disease	1			5

**Table 2:** Individual listing of patients. Reasons for revision arthroplasty and surgical interventions after joint puncture. PJI patients are highlighted in gray

Patient	Age (at time of surgery)	Gender	Reason for surgery	Intervention carried out (after joint puncture)
#1	63	F	PJI	THA explantation + antibiotic spacer
#2	88	F	PJI	THA explantation + antibiotic spacer
#3	79	M	PJI	TSA explantation + antibiotic spacer
#4	84	F	PJI	THA explantation + antibiotic spacer
#5	77	M	PJI	TKA explantation + antibiotic spacer
#6	76	F	PJI	THA explantation + antibiotic spacer
#7	76	F	PJI	THA explantation + antibiotic spacer
#8	63	F	PJI	TKA explantation + antibiotic spacer
#9	77	M	PJI	TKA explantation + antibiotic spacer
#10	81	M	PJI	TKA explantation + antibiotic spacer
#11	76	F	PJI	THA explantation + antibiotic spacer
#12	75	F	PJI	exchange of antibiotic spacer after THA explantation + antibiotic spacer
#13	79	F	PJI	THA explantation + antibiotic spacer
#14	55	F	Intraarticular free-floating cement-related particles after TKA	Removal of cement-related particles + inlay exchange
#15	79	M	Knee instability + scarred adhesions	Revision total knee arthroplasty
#16	45	F	Aseptic cup loosening	Revision hip arthroplasty of cup and femoral head
#17	79	F	Liner wear	Revision hip arthroplasty of femoral head and liner
#18	79	F	Recurrent hip dislocation	Revision hip arthroplasty of cup and femoral head
#19	51	M	Aseptic femoral and tibial loosening	Revision total knee arthroplasty
#20	81	F	Periprosthetic fracture	Revision total shoulder arthroplasty
#21	63	F	Spinal column associated pain, bursitis trochanterica	No revision, diagnostic joint aspiration of the hip (inset THA)
#22	80	F	Spinal column associated pain	No revision, diagnostic joint aspiration of the knee (inset TKA)
#23	69	F	Spinal column associated pain	No revision, diagnostic joint aspiration of the hip (inset THA)
#24	79	F	Recurrent patellar instability + arthrofibrosis	Arthrolysis + revision total knee arthroplasty
#25	72	M	Inlay wear, knee instability	Arthrolysis + inlay exchange
#26	43	F	Particle disease, aseptic peritrochanteric osteolysis	Exchange of femoral head and liner and bone void filling with synthetic CaP-cement
#27	83	M	Aseptic femoral and tibial loosening	Total revision knee arthroplasty
#28	82	F	Knee instability	Revision of femoral component + inlay exchange
#29	83	F	Periprosthetic fracture	Revision total shoulder arthroplasty
#30	81	F	Periprosthetic fracture	Revision total shoulder arthroplasty
#31	68	M	Lumboischialgia and hip pain	No revision, diagnostic joint aspiration (inset THA)
#32	78	F	Recurrent hip dislocations	Exchange of femoral head and inlay
#33	83	M	Aseptic femoral and tibial loosening	Total revision knee arthroplasty
#34	82	F	Knee instability	Revision of femoral component + inlay exchange
#35	38	F	Particle disease, aseptic osteolysis of the greater trochanter	Exchange of femoral head and inlay and bone void filling with CaP bone cement
#36	88	M	Aseptic femoral loosening	Revision of femoral stem, femoral head and liner
#37	52	F	Knee instability, inlay wear	Inlay exchange + partial patellar resection
#38	79	F	Aseptic acetabular loosening	Revision hip arthroplasty of cup

Of these 38 patients, 13 met the abovementioned criteria for PJI. There were six hips, six knees, and one shoulder. The PJI group included eight women and five men with a mean age of 76 years ± 8.5 (55-88). In this group, the synovial fluid of eleven patients was tested positive in the microbiological culture. Organisms include *Staphylcocci* (5×), *Klebsiella species* (3×), *Pseudomonas* (1×), *Cutibacterium acnes* (1×) and *Escherichia coli* (1×). One patient (8%) presented with a sinus tract. None of the patients were treated with antibiotics prior to culture sampling. Radiological signs of prosthetic loosening could be found in ten patients.

On the other side, 19 women and six men were included in the aseptic control group. There were 14 hips, nine knees, and two shoulders. In the control group, 15 patients suffered from polyethylene wear debris induced osteolysis. Fifteen patients (60%) received exchange of the polyethylene liner and head. Radiological signs of prosthetic loosening could be found and in ten patients. In two hips, corrosion of the modular head-neck junction could be found. Four patients suffered from spinal column associated pain and the diagnostic puncture of the hip (3× inset THA) or knee (1× inset TKA) was performed for differential diagnosis. Five patients of the control and one of the PJI group reported a systemic inflammatory disease before revision arthroplasty.

### Sample preparation and biomarker analysis

Synovial aspiration was performed under sterile conditions avoiding an admixture of blood with an 18-gage needle prior to surgical intervention.

Synovial fluid was aliquoted into sterile tubes and immediately put on ice. The synovial fluid samples were transported to the laboratory within 60 min and frozen and stored at −80 °C.

The immunoassay for synovial fluid BSP and synovial CRP was performed according to manufacturer’s specification using reagents from LifeSpan Bio (Seattle, USA). The specimens were measured in duplicates by standard enzyme-linked sandwich immunosorbent assay.

### Statistical analysis

The data was analyzed using SPSS Statistics 26 (IBM, Armonk, NY, USA). Descriptive statistics was used to analyze clinical and laboratory values. Statistical analysis indicated a non-parametric distribution of sBSP and sCRP in PJI and AF patients. Therefore, the Mann-Whitney *U* test was used to compare independent samples. For sensitivity, specificity and area under the curve (AUC) calculation, a receiver operating characteristics curve analysis was performed. *P* values below 0.05 were considered as statistically significant, values below 0.01 as highly significant.

## Results

### sBSP and sCRP behave in opposite ways

**Fig. 1:**
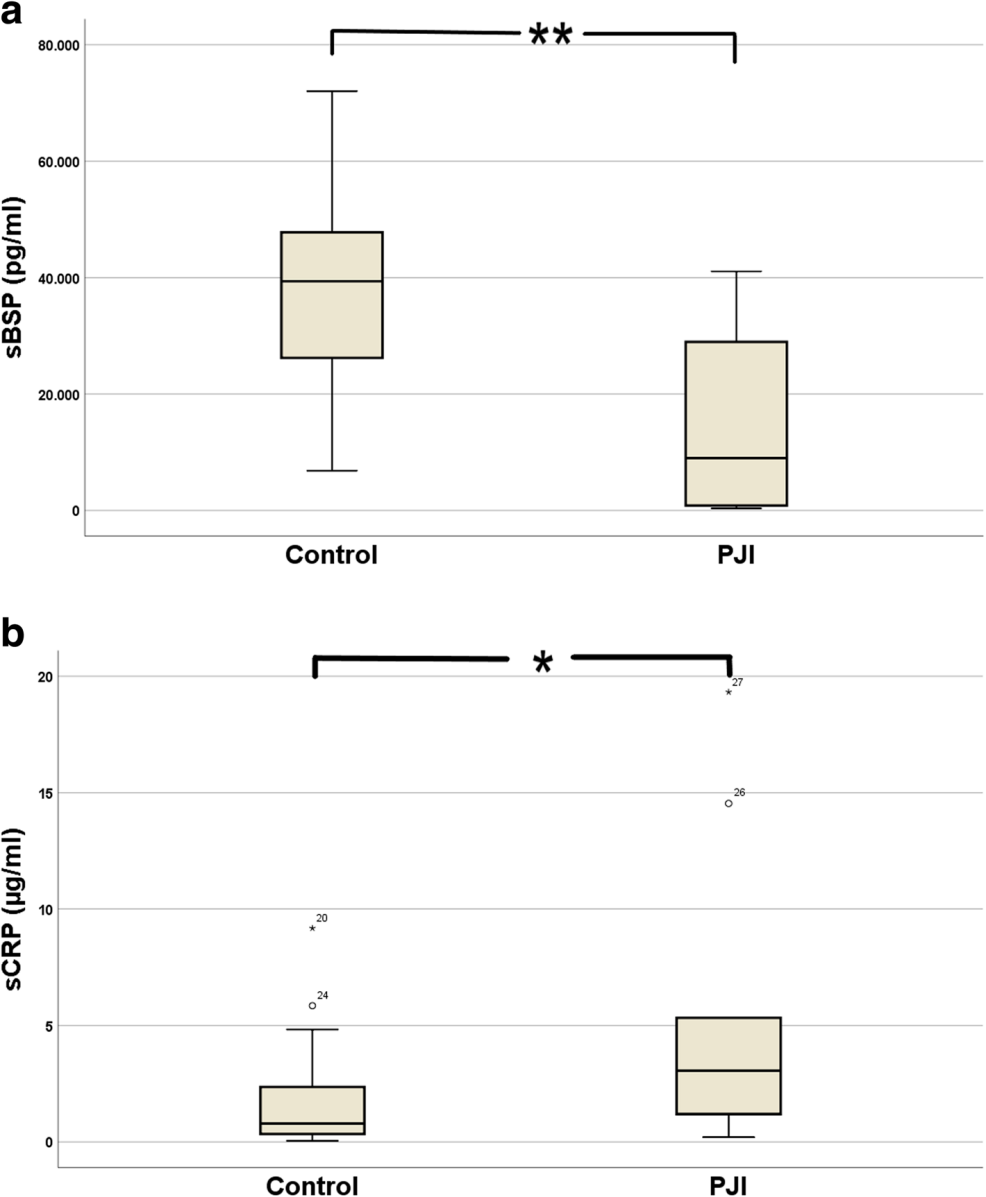
Boxplot diagram. Demonstrates the concentrations of sBSP in the control and PJI group (**a**) and of sCRP (**b**), respectively. The difference between the groups was highly significant in sBSP (**p ≤ 0.01) and significant in sCRP (*p ≤ 0.01)

The synovial bone sialoprotein and CRP results are presented in Fig. 1 (Fig. 1a, b). In patients with PJI according to the MSIS criteria, mean sBSP was significantly lower than in the aseptic group: 14.8 ng/ml (95% CI 5.5-24.1) vs. 38.2 ng/ml (95% CI 31.1-45.3), *p* ≤ 0.001. Conversely, mean sCRP was significantly higher in PJI patients: 8.4 μg/ml (95% CI 0-17.2) vs. 1.8 μg/ml in the control group (95% CI 0.9-2.8), *p* = 0.032.

In patients suffering from SID, mean sCRP was 3.3 μg/ml and in those who did not report SID it was 4.2 μg/ml (*p* = 0.80).

### Discrimination ability of sBSP and sCRP

**Fig. 2:**
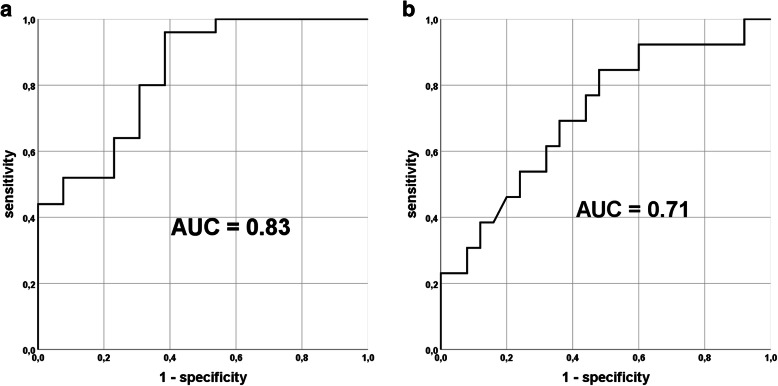
ROC analysis and AUC of synovial BSP (**a**) high sBSP in aseptic patients and CRP (**b**) high sCRP in PJI patients

The AUC of sBSP in aseptic revision arthroplasty patients was 0.83 (Fig. 2a). The AUC of sCRP in PJI patients was 0.71 (Fig. 2b). sBSP values of 24.4 ng/ml and above had a sensitivity of 0.69 and specificity of 0.80 in the detection of AF patients. The ROC curve analysis of sCRP revealed at a high specificity level of 0.92, a very poor test sensitivity of 0.31 (CRP threshold: 5.08 μg/ml).

### sBSP in joint nearby osteolyses, in *Staphylococci* induced PJI and systemic inflammatory disease

**Fig. 3:**
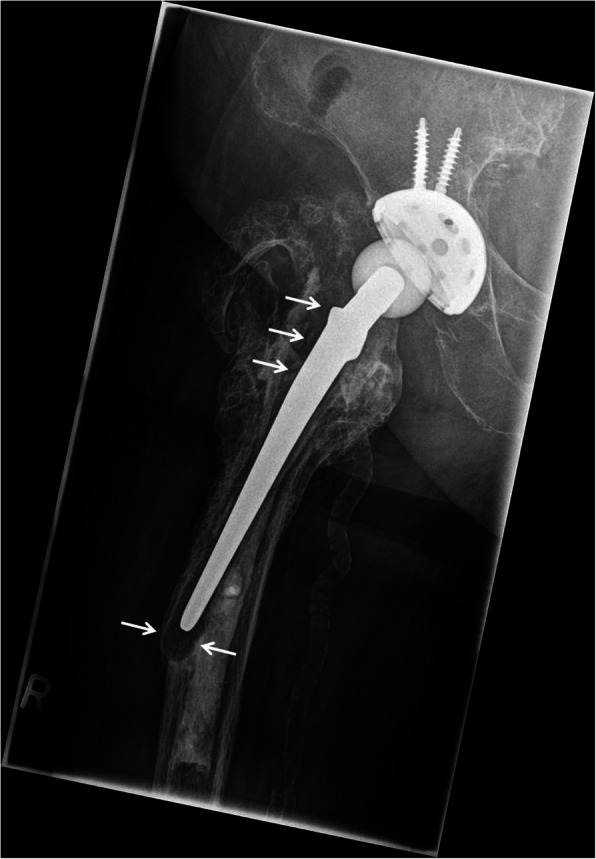
PJI of the right hip. Severe osteolyses of the cemented stem are visible (white arrows). *Pseudomonas aeruginosa* was detected in the synovial fluid. sBSP was measured low at 0.8 ng/ml and sCRP high at 14.5 μg/ml

As classic bone matrix protein, high sBSP values were speculated in synovial fluid of patients with nearby osteolyses due to an increased release from the bone matrix. In the PJI group, ten patients (77%) showed osteolytic lesions in conventional X-rays of the affected joint in two planes. In the control group, the rate of osteolytic lesions was 0.68 (*n* = 17). There was no significant difference of measured sBSP values between the detection of osteolyses (mean sBSP: 26.5 ng/ml) or not (vs. 39.3 ng/ml; *p* = 0.071). Fig. 3 demonstrates one case of a *Pseudomonas aeruginosa* PJI with severe osteolyses.

A tendency of higher mean sBSP values could be observed in *Staphylococci*-induced PJI compared to the group of other detected bacterial species (26.0 ng/ml vs. 4.2 ng/ml). This was a small sample comparison (*n* = 5 vs. *n* = 6) and the difference was not significant (*p* = 0.126).

No difference of sBSP values in SID compared to non-affected patients could be found (30.1 ng/ml vs. 30.2 ng/ml; *p* = 0.922).

## Discussion

Until today, the diagnosis of periprosthetic joint infections remains challenging. The latest proposed algorithm for diagnosing PJI by Parvizi et al. still includes, beside physical examination, the long-established serum C-reactive protein as a first step [18]. Recent research focused on novel serum or synovial biomarkers. Still, the aim of the search for suitable synovial biomarkers is to simplify PJI diagnostics.

Latest proteomic research of the synovial fluid identified lactoferrin, polymorphonuclear leukocyte serine protease 3, and myeloid nuclear differentiation antigen as potential candidates for reliable PJI diagnostics [19]. Others focused on synovial C-reactive protein, presepsin, or lipocalin-2 [5, 20, 21].

Against this backdrop, alpha-defensin was a promising candidate and is available on the market as a quick test. However, it is still not recommended in routine diagnostics, which might be a problem of its partially described poor sensitivity [7, 8, 18].

Test accuracy of all those investigated novel synovial biomarkers is calculated by using different established classification systems. These were proposed, inter alia, by various societies such as the musculoskeletal infection society (MSIS), the infectious diseases society of America or the European bone and joint infection society [7].

The newly proposed “2018 Definition of Periprosthetic Hip and Knee Infection” claims a nearly equal specificity but a noticeably higher sensitivity than the International Consensus Meeting definition or the MSIS, which was used in this study [2]. However, due to an effortful score calculation, it remains to be seen whether such a score will prevail in clinical routine. And especially in cases of a failed articular puncture, an intraoperative quick test of a synovial fluid biomarker may help to gain safety for the surgeon and the patient.

Remarkably, recent research did not focus on synovial markers to be elevated in aseptic failure TJA.

BSP is a significant component of the bone extracellular matrix and has been suggested to constitute approximately 8% of all non-collagenous proteins found in bone [22]. Binding of Staphylococci to BSP in osteomyelitis was firstly described by Rydén et al. in 1987 [12]. Not only *Staphylococcus aureus* but also *epidermidis* was identified to show this behavior [10, 23]. Further research revealed that an adhesion protein, named bone sialoprotein-binding protein, expressed by *Staphylococci* and able to bind the most abundant bone proteins may play a crucial role in orthopedic implant infections [24]. Due to bacterial binding of BSP in PJI patients, we speculated elevated synovial BSP in cases of AF patients and the results met our expectations. In the PJI cohort, on average lower BSP values were measured in synovial fluid samples. However, we could not find a connection of sBSP to concomitant local osteolyses detected in conventional radiographs. Nevertheless, this study does not answer the question if the reason for lower average sBSP values in PJI patients is bacterial binding of sBSP. This needs to be investigated in further studies.

Worldwide, serum CRP is a well-known diagnostic and process parameter in inflammation and commonly used in the diagnostic algorithm of PJI [2].

Synovial CRP was also measured in the samples of this study. According to the MSIS criteria, sCRP was fairly capable in distinguishing between the classes with an AUC of 0.71. In a recently published meta-analysis, synovial CRP was designated as a good biomarker for the diagnosis of PJI with high sensitivity and specificity [25]. The authors measured a pooled sensitivity of 0.92, a specificity of 0.90 and an AUC of 0.966. Our results do not support the described high accuracy of sCRP in the diagnostic of PJI. At a sensitivity level of 0.92 and above the sensitivity was only 0.31 which would have resulted in high amounts of false-positive test results at a threshold of 5.08 μg/ml sCRP in our cohort.

Other than sCRP or sBSP, alpha-defensin (sAD) is a microbicidal protein that is produced by neutrophils as a response to microbial products or proinflammatory cytokines. The promising results in its discrimination ability in PJI patients led to its increasing use in clinical practice. However, recent data support the fact of a high specificity but only poor sensitivity [8]. Our results of sCRP were not able to reach the good values of alpha-defensin. Even at a sensitivity level of in part just over 50%, sCRP was not able to reach a specificity of over 95% likewise alpha-defensin (Fig. 2b).

Interestingly, from an economic point of view, a recently published meta-analysis suggests that a simple leukocytes esterase strip may have the same power compared to a cost-intensive sAD measurement [26].

Five patients of our control group (20%) and one of the PJI patients (7.7%) suffered from systemic inflammatory diseases. Compared to the non-affected patients, no significant differences of sBSP or sCRP measurements could be found. This is in accordance to recent research that demonstrated that concomitant SID may not affect the accuracy of infection biomarkers in patients with PJI [27].

Future research should not only focus on novel synovial markers to detect PJI or AF patients but also on serum markers. This would solve the problem of failed joint punctures and it would significantly increase patient comfort. Because almost 100 years after the introduction of erythrocyte sedimentation rate and 90 years after the discovery of C-reactive protein, both are still state of the art in serum PJI diagnostics [28–30].

Limitations of this study are the exclusion of the sedimentation rate in the PJI diagnosis of our cohort. This may have had an impact on the sensitivity of the MSIS proceedings and at worst causing a higher proportion of false-negative results. Moreover, the MSIS criteria used in this study were published in 2011 and recently a validated updated version was published claiming a higher sensitivity than before [2]. Last but not least, the small sample size and low homogeneity of the included patients may have had a negative influence on the statistical power of our results. We advocate further studies with a more homogeneous patient population and especially the study of patients with suspected low-grade infections that do not clearly meet the MISIS criteria. It is precisely with these patients that synovial BSP could provide a benefit in everyday clinical practice.

## Conclusions

Considering the MSIS criteria, significantly higher sBSP concentrations were found in synovial fluid samples of AF compared to PJI patients. sCRP showed only fair, sBSP good discrimination potential. If it is not clear whether PJI is present or not, sBSP may be considered as an add-on synovial marker.

## Data Availability

The datasets analyzed during the current study are available from the corresponding author on reasonable request.

## Abbreviations

PJI: Periprosthetic joint infection
AF: Aseptic failure
TJI: Total joint arthroplasty
TSA: Total shoulder arthroplasty
THA: Total hip arthroplasty
TKA: Total knee arthroplasty
sBSP: Synovial bone sialoprotein
sCRP: Synovial C-reactive protein
MSIS: Muskuloskeletal infection society
AUC: Area under the curve
Bbp: Bone sialoprotein-binding protein
IRB: Institutional Review Board
SID: Systemic inflammatory disease
sAD: Synovial alpha-defensin
CaP: Calcium phosphate
